# Supplementary material for: Dysregulation of mitochondrial dynamics proteins are a targetable feature of human tumors
Source: Nat Commun. 2018 Apr 26;9:1677. doi: 10.1038/s41467-018-04033-x (PMC5919970; doi:10.1038/s41467-018-04033-x)
Supplement: Supplementary file 1 — Supplementary Information [file 41467_2018_4033_MOESM1_ESM.pdf]

Dysregulation of mitochondrial dynamics proteins are a targetable feature of human tumors

Anderson et al.

Supplementary Information

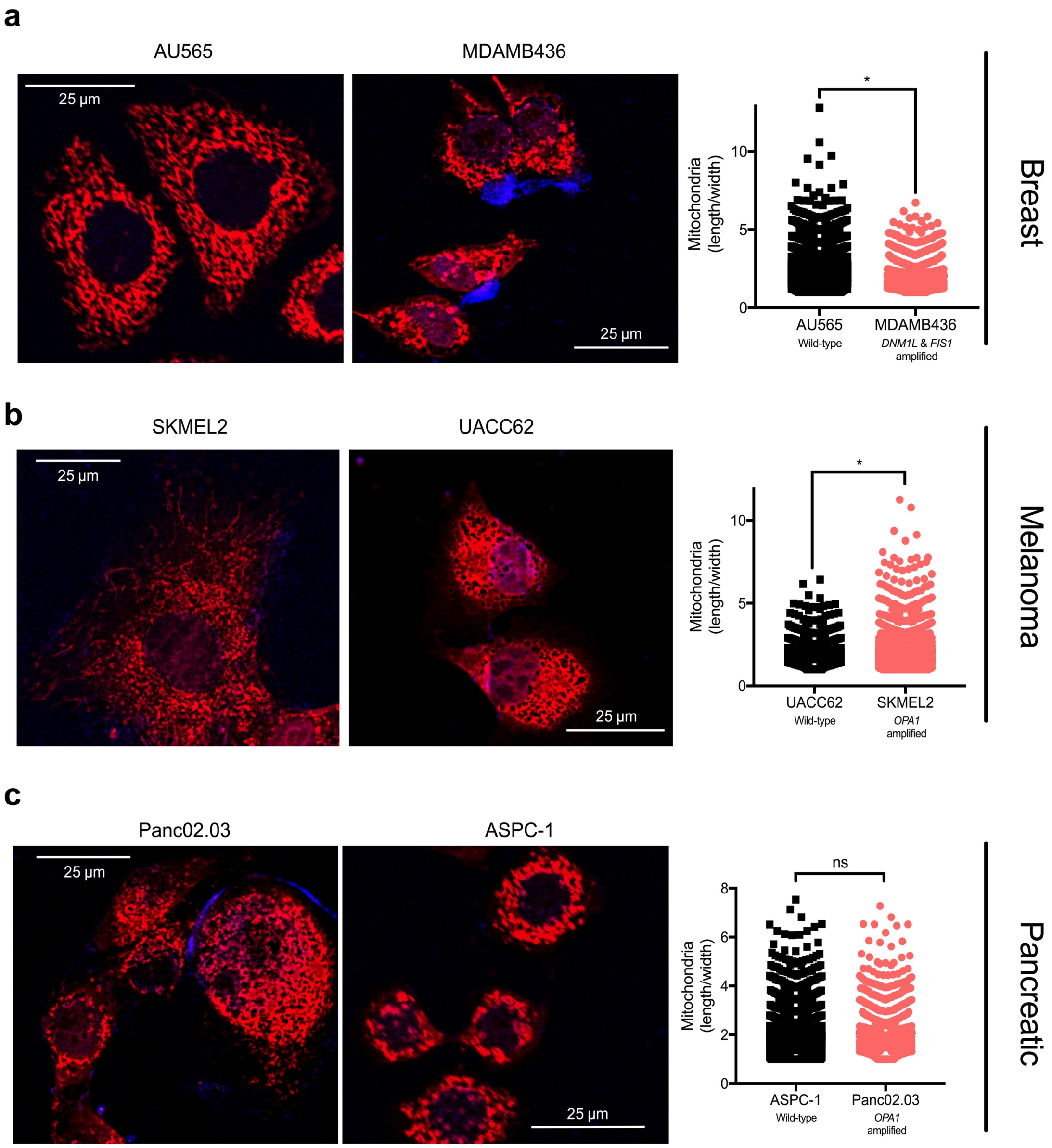

Supplementary Figure 1: Amplifications in dynamics regulating genes can results in commiserate changes to mitochondrial morphology. a) Mitotracker and DAPI in two breast cancer cell lines AU565 (wild-type) and MDAMB436 (*DNM1L* and *FIS1* amplifications). Right, mitochondrial length x width quantification is plotted for thousands of mitochondria from >10 cells across at least two independent experiments. *$p < 0.05$ by student's t-test. **b)** Mitotracker and DAPI in two melanoma cancer cell lines SKMEL2 (*OPA1* amplified) and UACC62 (wild-type). Right, mitochondrial length x width quantification is plotted for thousands of mitochondria from

>10 cells across at least two independent experiments. *$p < 0.05$ by student's t-test. **c)** Mitotracker and DAPI in two pancreatic cancer cell lines Panc02.03 (*OPA1* amplified) and ASPC-1 (wild-type). Right, mitochondrial length x width quantification is plotted for thousands of mitochondria from >10 cells across at least two independent experiments. *$p < 0.05$ by student's t-test. For a-c, scale bars 25 μm and immunofluorescence images are representative of two independent experiments.

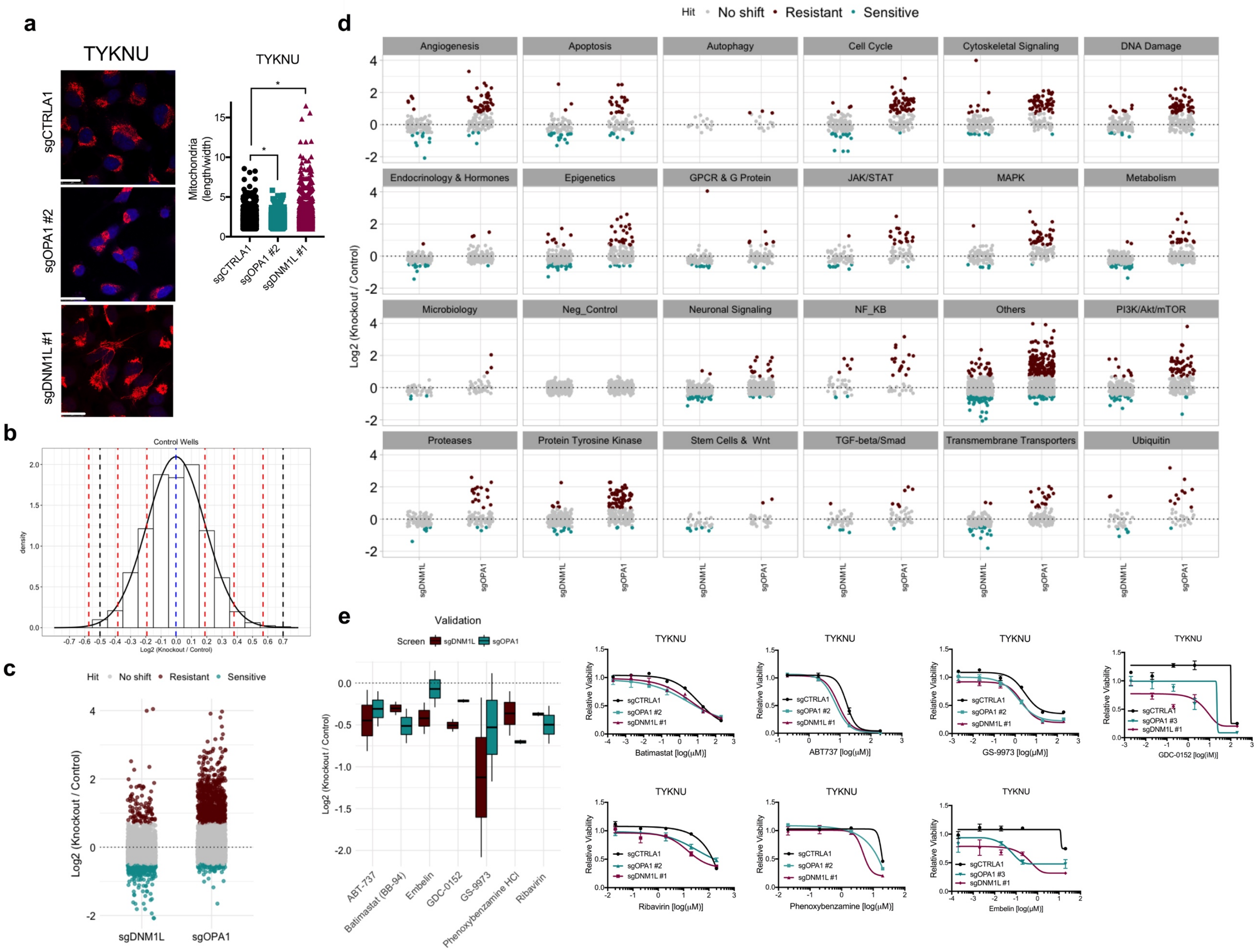

Supplementary Figure 2: Small-molecule screening results and validation of screening hits. a) Mitotracker red and DAPI staining on TYKNU cells transduced with a control sgRNA or a representative sgRNA targeting either *OPA1* or *DNM1L*. Scale bars 25 μm. Immunofluorescence images are representative of two independent experiments. Right, mitochondrial length x width quantification is plotted for thousands of mitochondria from >10 cells across at least two independent experiments. *p < 0.05 by one-way anova. **b)** Density plot of the distribution of the Log_2(knockout/control) viability ratios of the control wells. Blue dotted line indicates the mean value. Each set of red dotted lines indicates 1 SD away from the mean. The

black dotted line indicates the threshold for hit calling. **c)** The Log_2(knockout/control) ratio in both doses of the small-molecule library. **d)** The Log_2(knockout/control) ratio binned into the pathway annotation for each drug. **e)** Left, Log_2(knockout/control) ratio for seven drugs that were called as hits in the small-molecule screen. Right, secondary validation GI_{50} curves for each of the drugs from the left panel in TYKNU cells transduced with a control sgRNA and a representative sgRNA targeting either *DNM1L* or *OPA1* (n=3).

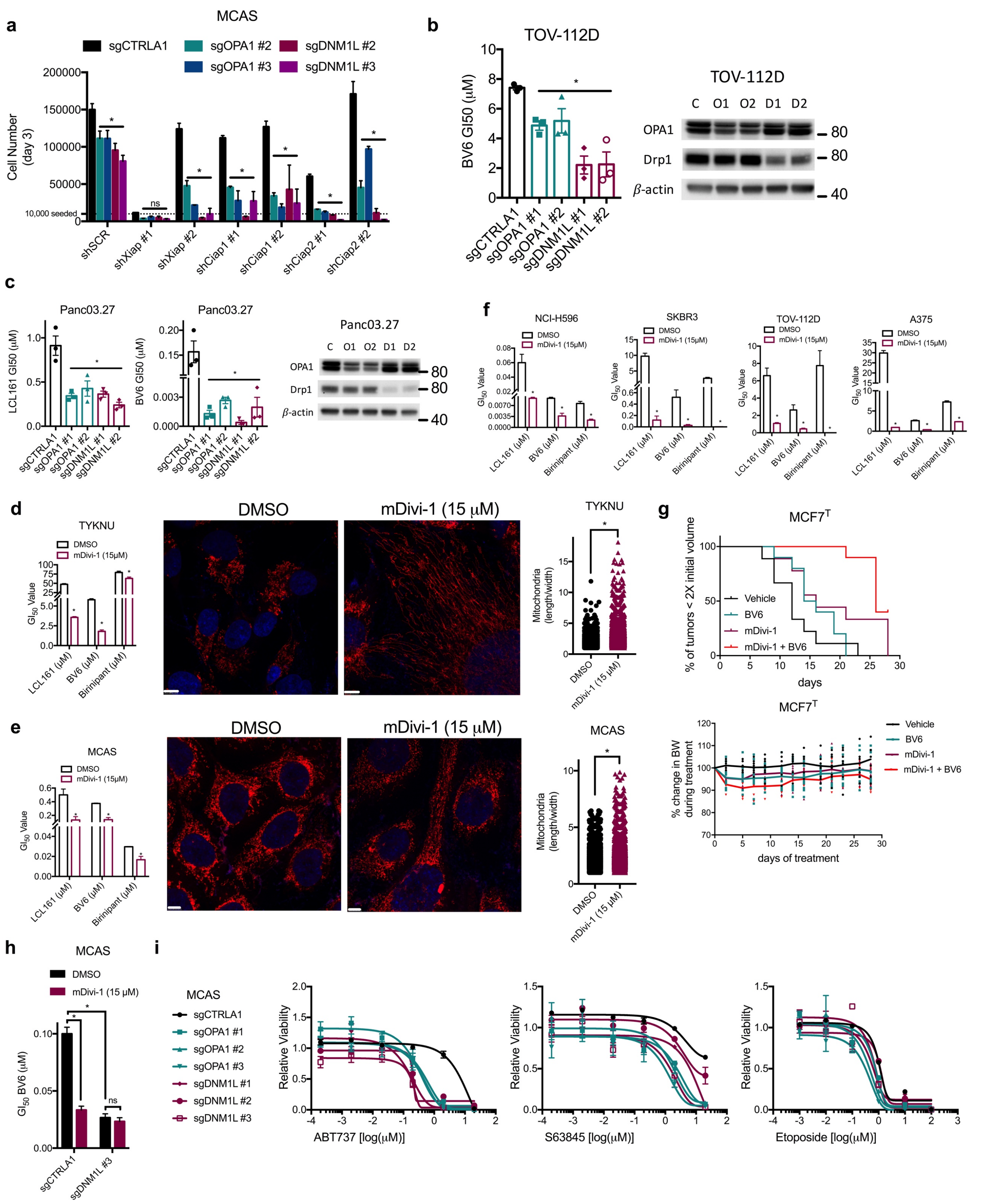

Supplementary Figure 3: Genetic and pharmacological validation of increased potency of

SMAC mimetics across cancer tissue types. a) 2D growth assay plotted as cell number in MCAS cells transduced with one of the following: a control targeting sgRNA, one of two *OPA1* targeting sgRNAs, or one of two *DNM1L* targeting sgRNAs—and one of the following: a scramble shRNA, or one of two XIAP targeting shRNAs, or one of two CIAP1 targeting shRNAs, or one of two CIAP2 targeting shRNAs. (n=3) Data are mean ± SEM. *p < 0.05 by one-way ANOVA. **b)** Left, GI_{50} values for BV6 in TOV-112D cells transduced with a control targeting sgRNA, or two independent sgRNAs targeting either *OPA1* or *DNM1L* (n=3). Data are mean ± SEM. *p < 0.05 by one-way ANOVA. Right, immunoblot of *OPA1* or *DNM1L* in cells from the middle. **c)** Left, GI_{50} values for LCL161 and BV6 in Panc03.27 cells transduced with a control targeting sgRNA or two independent sgRNAs targeting either *OPA1* or *DNM1L* (n=3). Data are mean ± SEM. *p < 0.05 by one-way ANOVA. Right, immunoblot of *OPA1* or *DNM1L* in cells from the left. **d)** Left, GI_{50} values for three SMAC mimetics (LCL161, BV6, Birinipant) in TYKNU cells treated with either vehicle or mDivi-1 (15 μM). Data are mean ± SEM. * p < 0.05 by one-way ANOVA. Right, mitotracker red and DAPI staining in TYKNU cells treated with vehicle or mDivi-1 (15 μM). Scale bars 7.5 μm. Immunofluorescence images are representative of two independent experiments. **e)** Left, GI_{50} values for three SMAC mimetics (LCL161, BV6, Birinipant) in MCAS cells treated with either vehicle or mDivi-1 (15 μM). Data are mean ± SEM. * p < 0.05 by one-way ANOVA. (n=3). Right, mitotracker red and DAPI staining in MCAS cells treated with vehicle or mDivi-1 (15 μM) (scale bars 7.5 μm). Immunofluorescence images are representative of two independent experiments. **f)** GI_{50} values for three SMAC mimetics (LCL161, BV6, Birinipant) in NCIH596, SKBR3, TOV-112D, or A375 cells treated with either vehicle or mDivi-1 (15 μM) (n=3). Data are mean ± SEM. * p < 0.05 by one-way ANOVA. **g)** Top, survival plot of *in vivo* data from Figure 3e as the number of mice with tumors less than 2X the original size (see methods for n in each

group and statistics). Right, weights of the mice from *in vivo* data in Figure 3e (see methods for n in each group and statistics). Immunoblots are cropped for clarity. **h)** GI_{50} values to BV6 in MCAS cells transduced with either a control targeting sgRNA or a *DNM1L* targeting sgRNA and treated with either vehicle or mDivi-1 (15 μM) (n=3). Data are mean ± SEM. * $p < 0.05$ by one-way ANOVA. **i)** GI_{50} curves for MCAS cells transduced with either a control targeting sgRNA or three independent sgRNAs targeting *DNM1L* or *OPA1* for three drugs (ABT737, S63845, Etoposide) (n=3). Data are mean ± SEM.

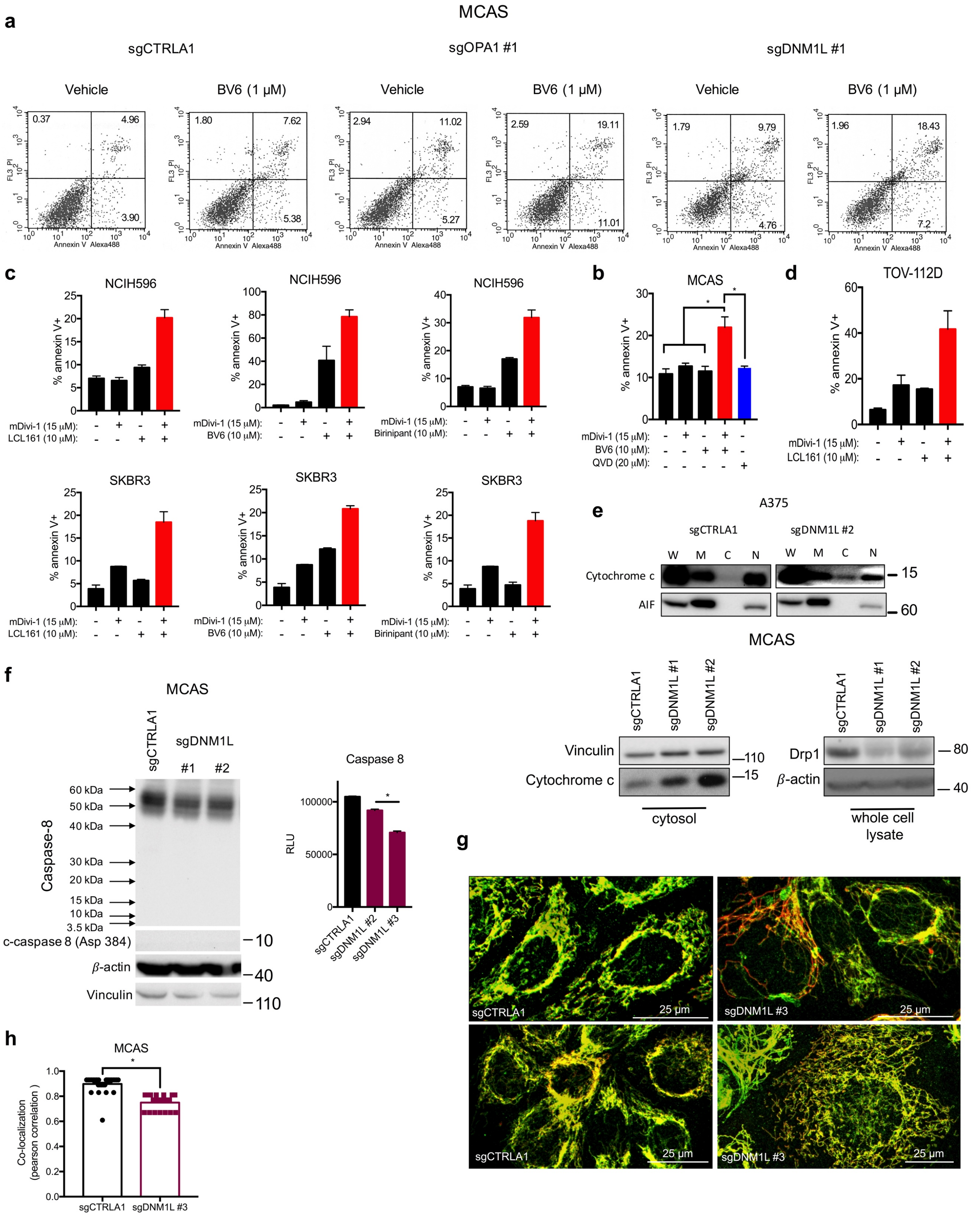

Supplementary Figure 4: Mechanism of sensitivity to SMAC mimetics in *DNM1L* loss

cells. a) Annexin V and PI staining for MCAS cells transduced with either a control targeting sgRNA, a *DNM1L* targeting sgRNA, or an *OPA1* targeting sgRNA treated with vehicle or BV6 (1 μM). **b)** Percentage of annexin V+ cells following treatment with vehicle, mDivi-1, BV6, the combination, or the combination plus Q-VD-OPh (a pan caspase inhibitor) (n=3). Data are mean ± SEM. *$p < 0.05$ by one-way ANOVA. **c)** Top, percentage of annexin V+ cells following treatment with vehicle, mDivi-1, SMAC mimetic (BV6, LCL161, or Birinipant), or the combination in NCIH596 cells (n=3). Data are mean ± SEM. *$p < 0.05$ by one-way ANOVA. Bottom, percentage of annexin V+ cells following treatment with vehicle, mDivi-1, SMAC mimetic (BV6, LCL161, or Birinipant), or the combination in SKBR3 cells (n=3). Data are mean ± SEM. *$p < 0.05$ by one-way ANOVA. **d)** Percentage of annexin V+ cells following treatment with vehicle, mDivi-1, LCL161, or the combination in TOV-112D cells (n=3). Data are mean ± SEM. *$p < 0.05$ by one-way ANOVA. **e)** Top**,** immunoblot of cytochrome c or AIF in whole cell lysate, membrane fraction, cytosolic fraction, and nuclear fraction of A375 cells transduced with a control sgRNA or a representative sgRNA targeting *DNM1L.* Bottom, immunoblot of vinculin and cytochrome c (cytosolic fraction), and Drp1 and β-actin (whole cell lysate), in MCAS cells transduced with control sgRNA or two independent sgRNAs for *DNM1L*. Immunoblots are representative of two independent experiments. AIF is used as a control for contamination of the cytosolic fraction. **f)** Left, immunoblot of vinculin, β-actin, T-caspase 8, and c-caspase 8 in MCAS cells transduced with a control sgRNA or two independent sgRNAs for *DNM1L*. Immunoblots are representative of two independent experiments. Right, raw luminescence units of caspase 8 activity in MCAS cells transduced with control sgRNA or two independent sgRNAs for *DNM1L* (n=3). Data are mean ± SEM. *$p < 0.05$ by one-way ANOVA. **g)** Tom20 (red) and cytochrome c (green) immunofluorescence in MCAS cells transduced with a control sgRNA or *DNM1L* targeting

sgRNA. Scale bar is 25 μm. Immunofluorescence images are representative of two independent experiments. **h)** Co-localization (Pearson correlation) quantification of two different image channels in at least 25 cells across at least two independent experiments. Higher correlation values indicate a greater extent of co-localization. *$p < 0.05$ by student's t-test.

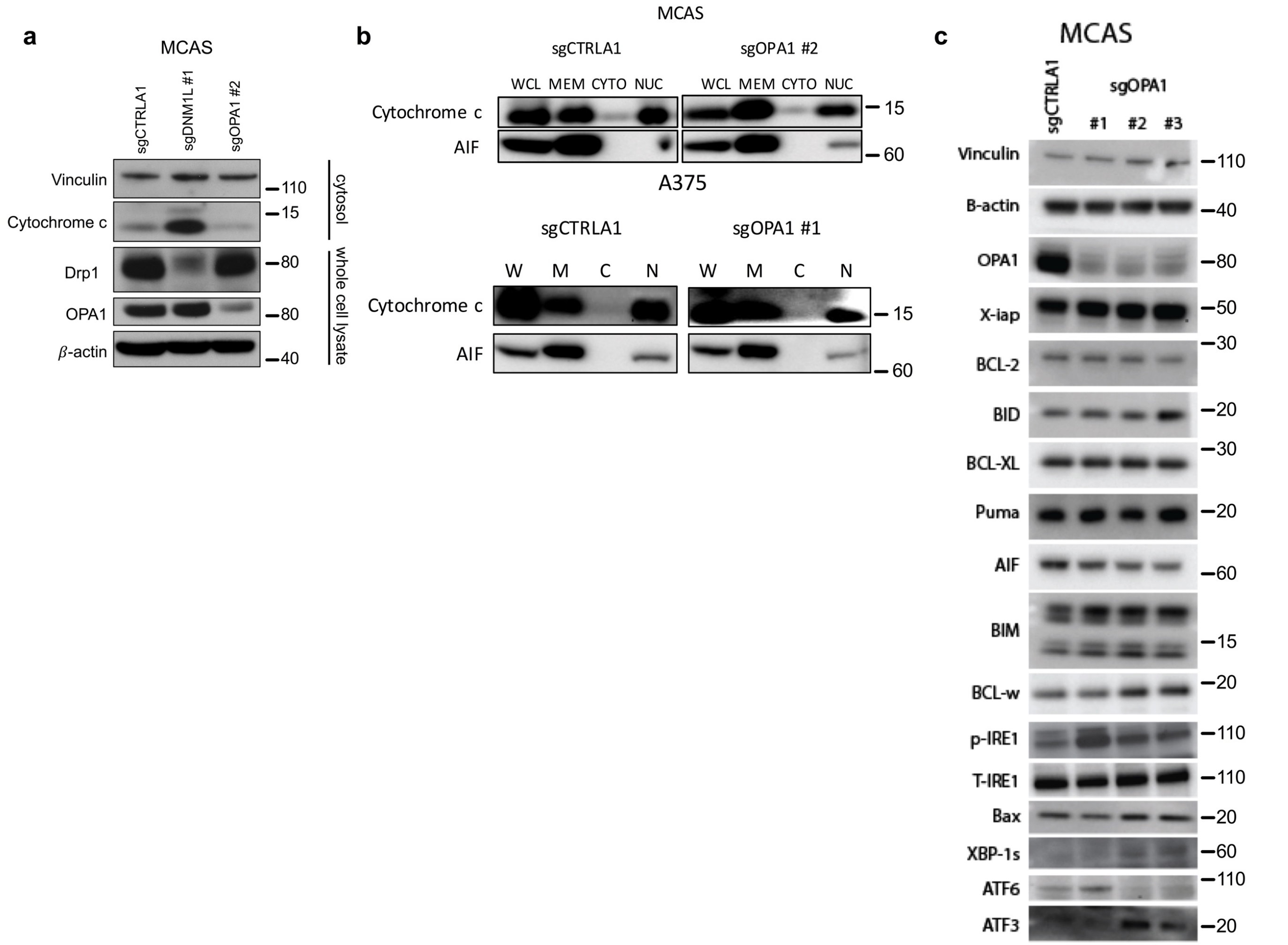

Supplementary Figure 5: Mechanism of sensitivity to SMAC mimetics in *OPA1* loss cells.

a) Immunoblot of indicated proteins in cytosolic fraction or whole cell lysate of MCAS cells transduced with a control sgRNA and representative sgRNAs for *DNM1L* and *OPA1*. Immunoblots are representative of two independent experiments. **b)** Top**,** Immunoblot of cytochrome c or AIF in whole cell lysate, membrane fraction, cytosolic fraction, and nuclear fraction of MCAS cells transduced with a control sgRNA or a representative sgRNA targeting *OPA1.* Bottom, same as above panel but in A375 cells. Immunoblots are representative of two independent experiments. AIF is used as a control for fractionation, as it should be present only in the membrane fraction. **c)** Immunoblot of indicated proteins in MCAS cells transduced with a

control sgRNA and three sgRNAs targeting *OPA1*. Immunoblots are cropped for clarity. Immunoblots are representative of two independent experiments.

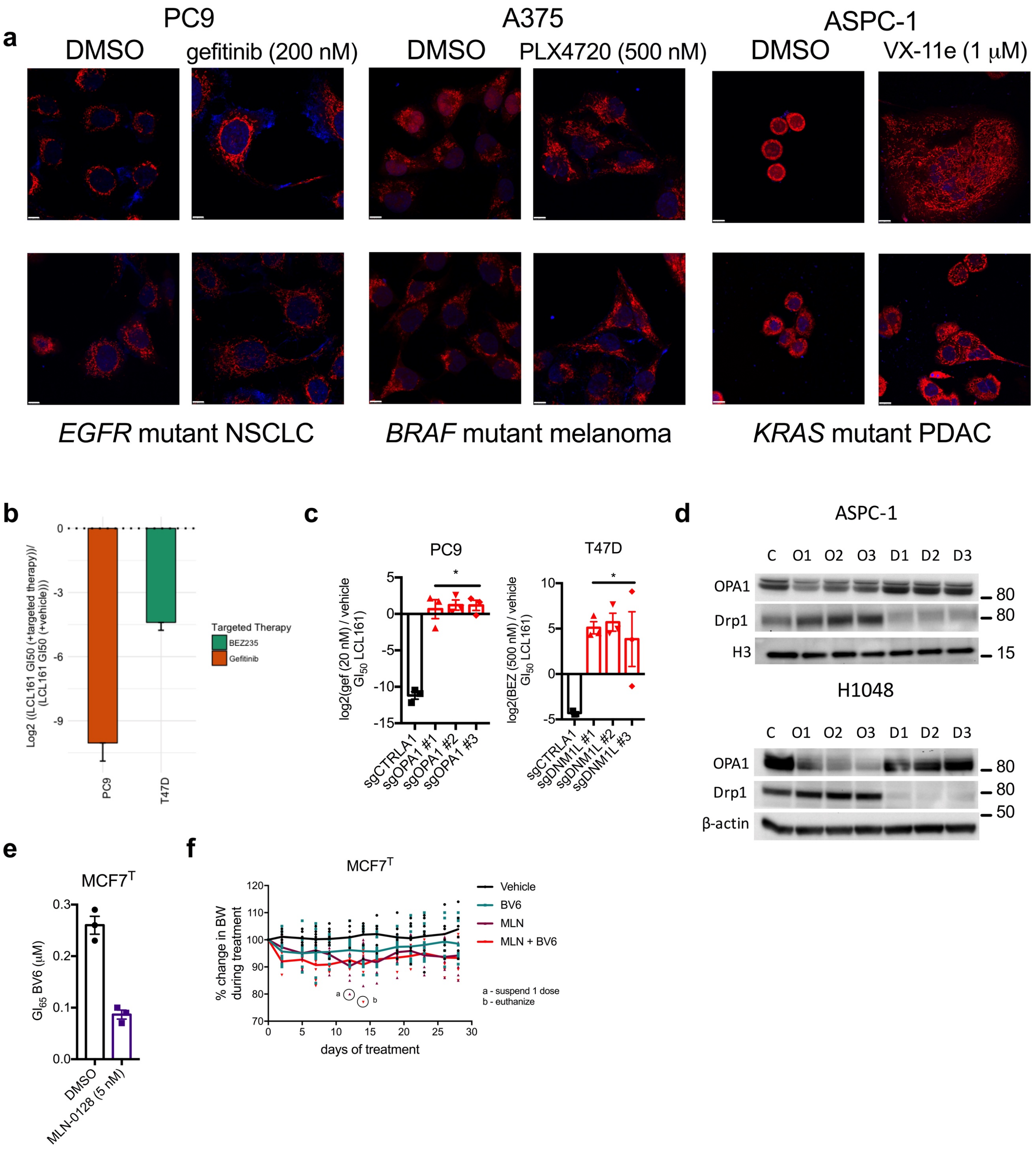

Supplementary Figure 6: Oncogenic control of mitochondrial dynamics and sensitivity to SMAC mimetics. a) Mitotracker and DAPI in three cell lines: PC9 (scale bars 7.5 μm), A375

(scale bars 7.5 μm), ASPC-1 (scale bars 10 μm) treated with vehicle or one of three inhibitors (gefitinib, PLX4720, VX-11e). Immunofluorescence images are representative of two independent experiments. **b)** Log_2((LCL161 GI50 (+targeted therapy))/(LCL161 GI50 (+vehicle))) in two cell lines driven by different oncogenes (n=3). Data are mean ± SEM. **c)** Log_2((LCL161 GI50 (+targeted therapy))/(LCL161 GI50 (+vehicle))) in two cell lines driven by different oncogenes transduced with a control sgRNA, three sgRNAs targeting *DNM1L,* or three sgRNAs targeting *OPA1* (n=3). Data are mean ± SEM. *p < 0.05 by one-way ANOVA. **d)** Top, Immunoblot of OPA1 and Drp1 in ASPC-1 cells transduced with a control sgRNA or three sgRNAs targeting either *DNM1L* or *OPA1*. Bottom, same as above but in H1048 cells. Immunoblots are representative of two independent experiments. **e)** GI_{65} to BV6 in $MCF7^T$ cells treated with either vehicle or MLN-0128 (5 nM) (n=3). Data are mean ± SEM. *p < 0.05 by student's t-test. **f)** Weights of the mice from *in vivo* data in Figure 6d (see methods for n in each group and statistics).

Figure 2a

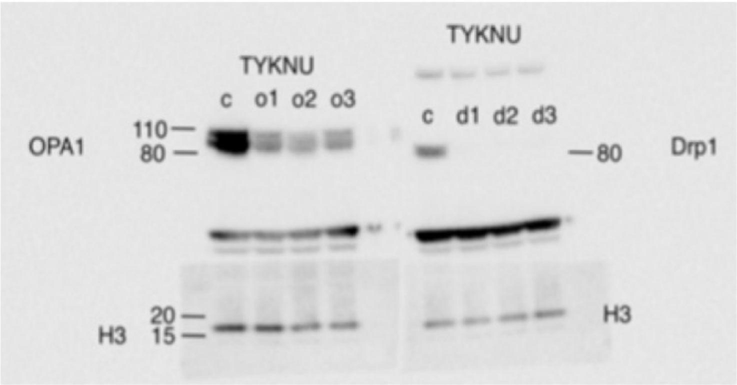

Figure 3c

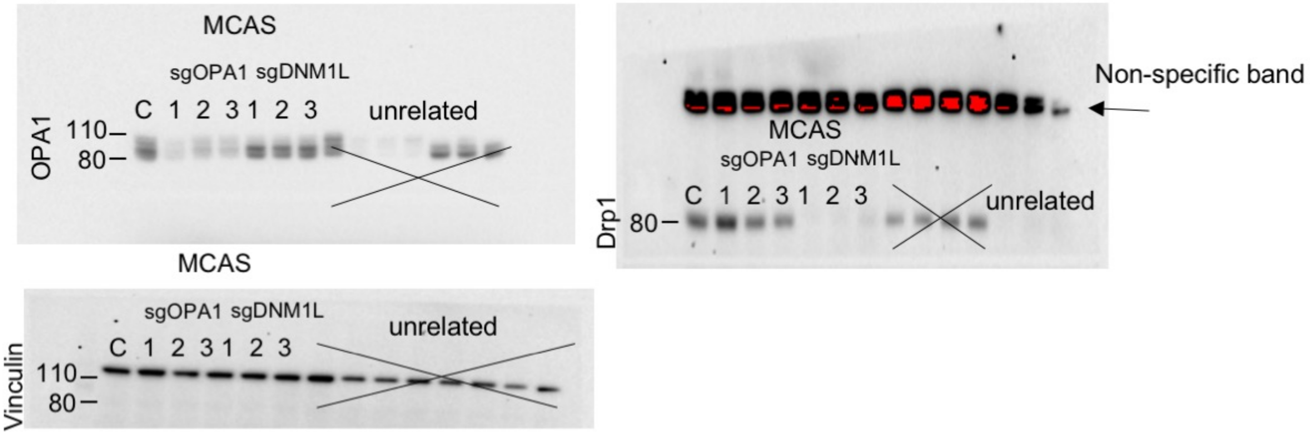

Figure 3d,e

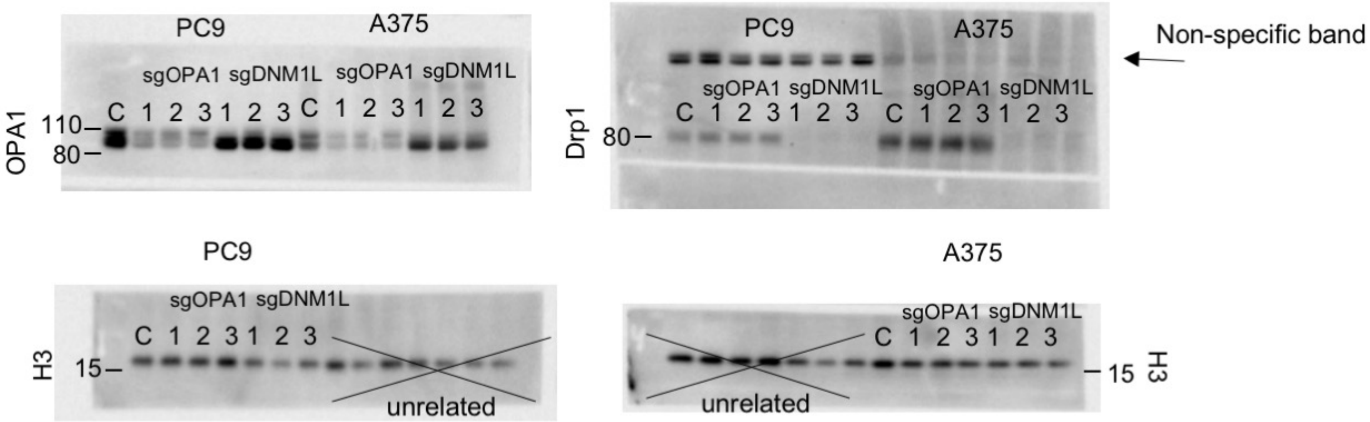

Supplementary Figure 7: Uncropped westerns for various figures.

Figure 3f

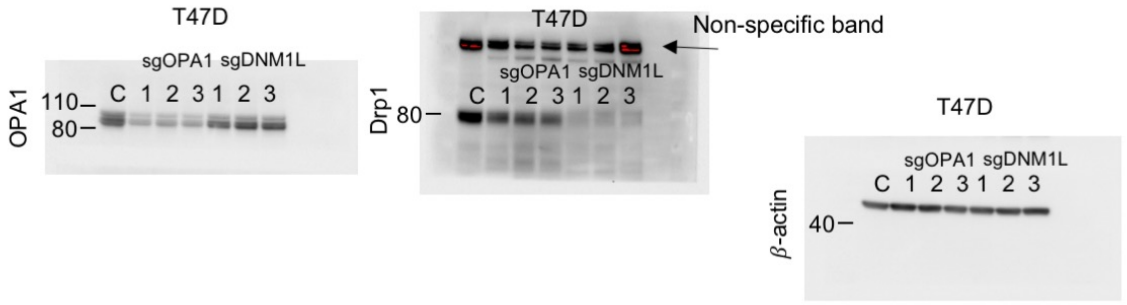

Figure 4b

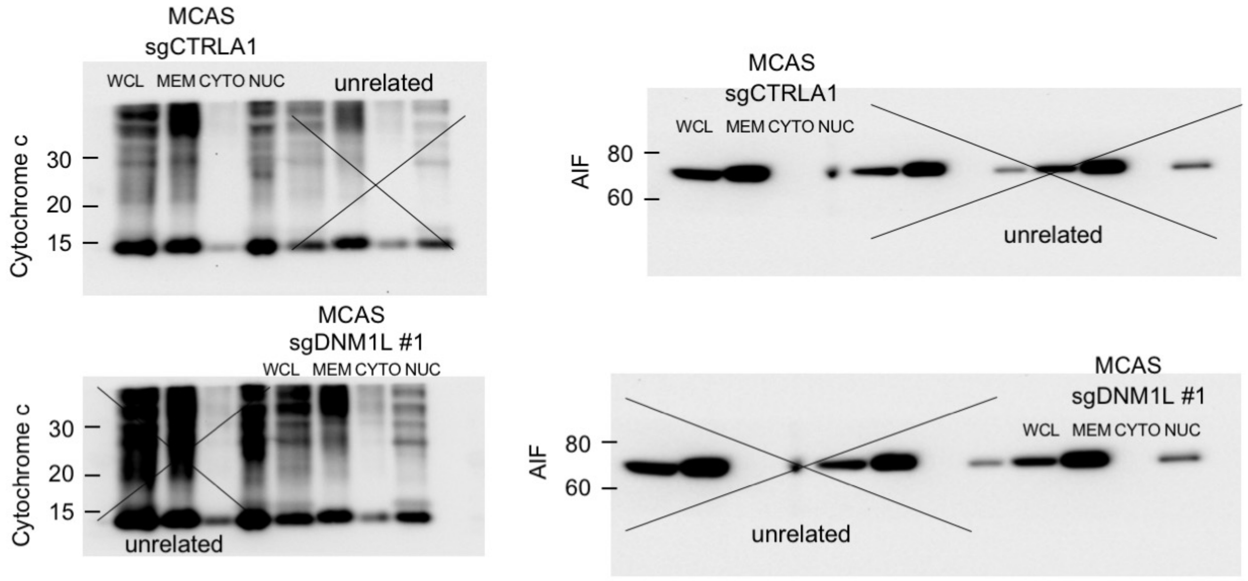

Figure 4c

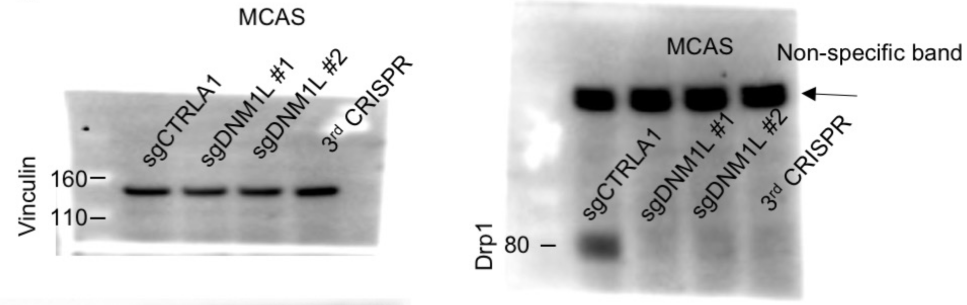

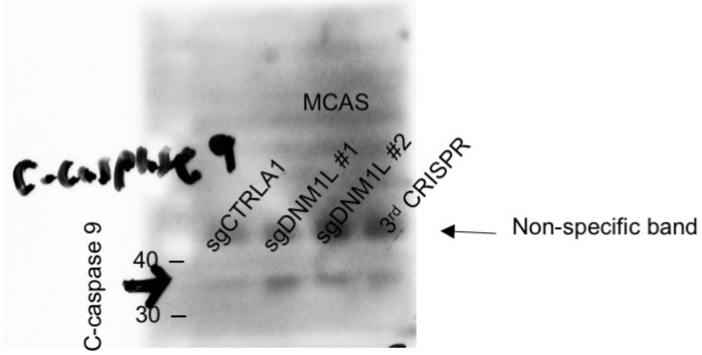

3rd CRISPR is not in the published manuscript

Supplementary Figure 7: Uncropped westerns for various figures.

Figure 5d

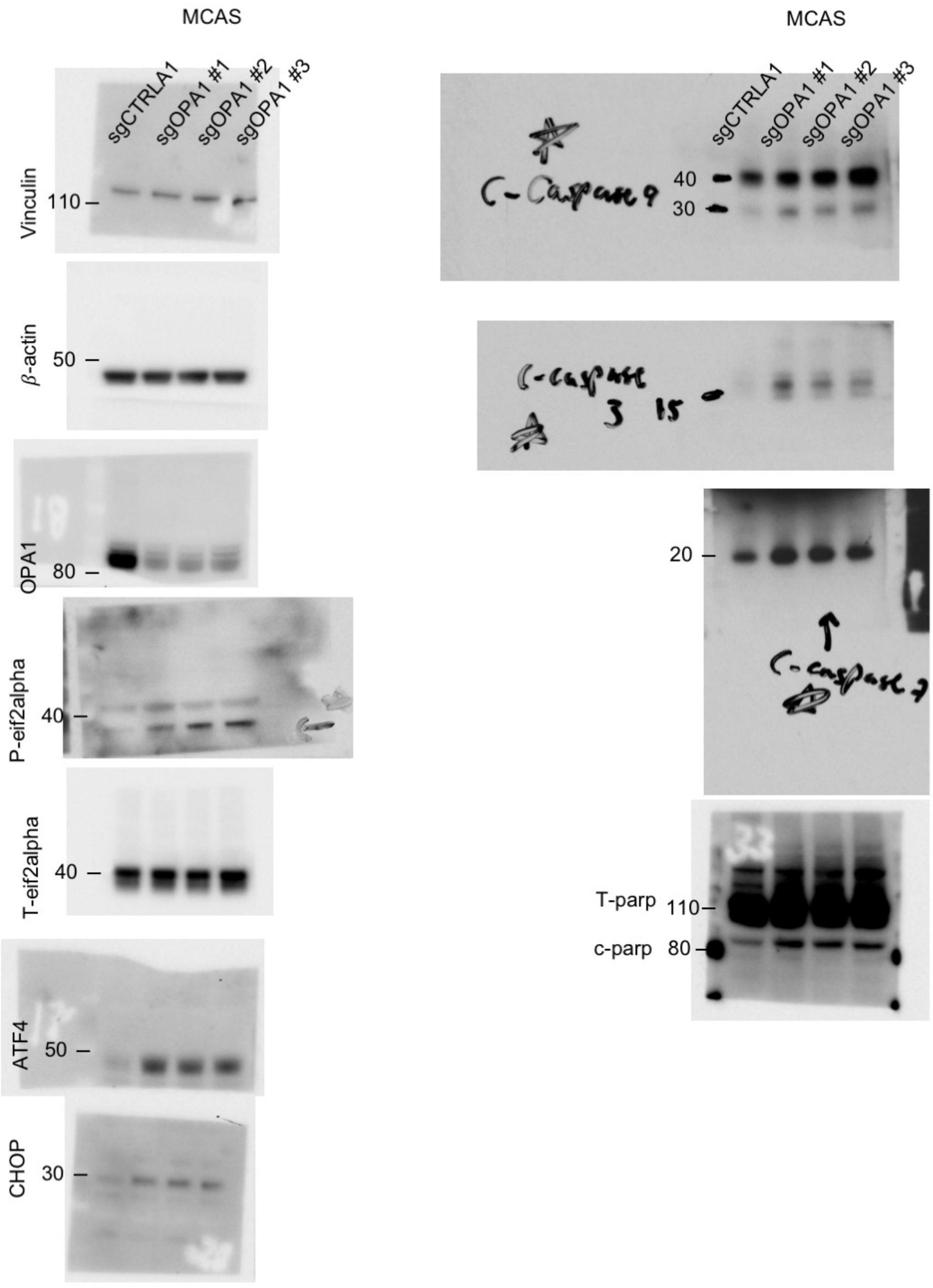

Supplementary Figure 7: Uncropped westerns for various figures.

Figure 5h

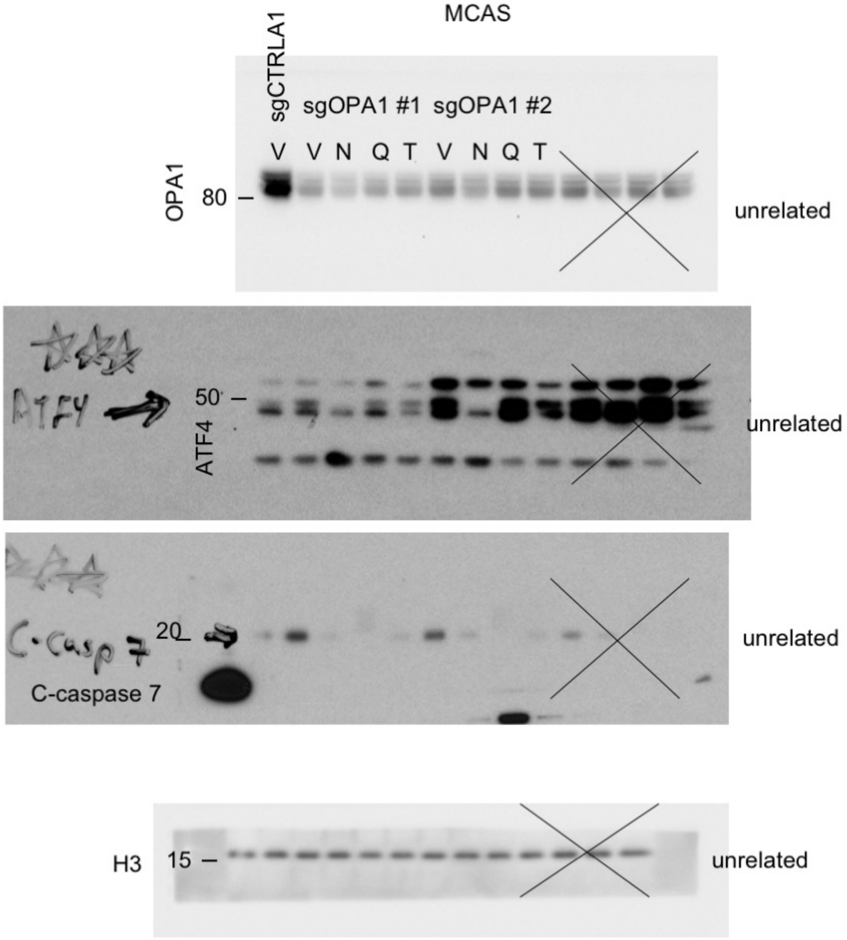

Supplementary Figure 7: Uncropped westerns for various figures.
