# Supplementary material for: Dysregulation of mitochondrial dynamics proteins are a targetable feature of human tumors
Source: Nat Commun. 2018 Apr 26;9:1677. doi: 10.1038/s41467-018-04033-x (PMC5919970; doi:10.1038/s41467-018-04033-x)
Supplement: Supplementary file 3 — Description of Additional Supplementary Files [file 41467_2018_4033_MOESM3_ESM.pdf]

Description of Additional Supplementary Files

File Name: Supplementary Data 1
Description: Computational identification of drugs with differential potencies in cell lines with alterations in mitochondrial dynamics-regulating genes.

File Name: Supplementary Data 2
Description: Screening data for DNM1L and OPA1 knockout derivatives.
